# Deep Profiling of the Aggregated Proteome in Alzheimer’s Disease: From Pathology to Disease Mechanisms

**DOI:** 10.3390/proteomes6040046

**Published:** 2018-11-12

**Authors:** Brianna M. Lutz, Junmin Peng

**Affiliations:** Departments of Structural Biology and Developmental Neurobiology, Center for Proteomics and Metabolomics, St. Jude Children’s Research Hospital, Memphis, TN 38105, USA; Brianna.lutz@stjude.org

**Keywords:** proteomics, proteome, mass spectrometry, Alzheimer’s disease, protein aggregation, laser capture microdissection, splicing, U1 snRNP

## Abstract

Hallmarks of Alzheimer’s disease (AD), a progressive neurodegenerative disease causing dementia, include protein aggregates such as amyloid beta plaques and tau neurofibrillary tangles in a patient’s brain. Understanding the complete composition and structure of protein aggregates in AD can shed light on the as-yet unidentified underlying mechanisms of AD development and progression. Biochemical isolation of aggregates coupled with mass spectrometry (MS) provides a comprehensive proteomic analysis of aggregates in AD. Dissection of these AD-specific aggregate components, such as U1 small nuclear ribonucleoprotein complex (U1 snRNP), provides novel insights into the deregulation of RNA splicing in the disease. In this review, we summarize the methodologies of laser capture microdissection (LCM) and differential extraction to analyze the aggregated proteomes in AD samples, and discuss the derived novel insights that may contribute to AD pathogenesis.

## 1. Introduction

Alzheimer’s Disease (AD) is a progressive neurodegenerative disease and the most common form of dementia, listed as the sixth leading cause of death [1,2]. AD represents a major economic burden predicted to surpass one trillion dollars worldwide in 2018 [3]. The cause of AD, however, is still not fully understood. There is no cure for AD, and current therapeutic strategies cannot hinder cognitive decline in AD [4].

The pathogenesis of AD has been extensively investigated by genetic and biochemical approaches. Genetic analysis of AD patients established three causative genes (*APP*, *PSEN1* and *PSEN2*) and a high-risk allele (*ApoE ε4*) [5,6], whereas genome-wide association studies led to the discovery of more than 20 low-risk genetic loci [7,8,9], and more recently, high-throughput sequencing identified rare, medium-risk genes, such as *TREM2* [10] and *UNC5C* [11]. Despite the genetic contributions, the vast majority of AD cases are sporadic, which may be attributed to the combination of genetic susceptibility and environmental factors [5], such as Herpesvirus infection [12,13] and environmental pollutants [14]. Biochemical dissection of AD brain tissue identified pathological hallmarks of amyloid-β (Aβ)-containing amyloid plaques, and neurofibrillary tangles (NFT) comprising hyperphosphorylated Tau in both familial and sporadic patients [15], although Tau mutations were identified in other forms of dementia, collectively termed tauopathy [16]. These results lead to the proposed amyloid cascade and Tau hypotheses [16,17] dominating AD research.

In the amyloid cascade and Tau hypotheses, the accumulation of Amyloid Precursor Protein (APP)-derived Aβ peptide is assumed to be the main cause of AD. Toxic Aβ species in the brain trigger a cascade that leads to inflammation, tau hyperphosphorylation and deposition, synaptic loss and neuronal degeneration, which eventually leads to dementia in AD. Based on the hypotheses, numerous animal models (largely mouse models) have been developed to mimic some phenotypes observed in AD patients, but these models cannot fully recapitulate human AD symptoms [18,19].

In addition, there is a lack of concordance between these models and clinical trials [18,19]. Given the amyloid hypothesis, targeting the cleavage of APP or the accumulation of Aβ has long been a goal for a pharmacological treatment for AD [20]. Unfortunately, clinical trials implementing Aβ antibody therapy or pharmacological intervention of APP cleavage have not yet been successful [21,22]. There is an urgent need for a broad understanding of synergistic interactions of molecular and cellular components in the brain, at asymptomatic—when the pathological hallmarks of AD are present but cognitive dysfunction is not evident [23]—and symptomatic stages during AD progression [24].

We believe that deep analysis of protein deposition in AD has the potential to discover novel disease mechanisms, considering the profound impact of the previous identification of Aβ and tau aggregation on our understanding of AD. In addition, protein aggregation is commonly observed in other neurodegenerative disorders, such as α-synuclein in Parkinson disease (PD) [25], and TDP-43 in ubiquitin-positive frontotemporal lobar degeneration (FTLD-U) and amyotrophic lateral sclerosis (ALS) [26]. In this review, we summarize the approaches toward profiling protein aggregates in AD, with a discussion of the benefits and pitfalls of the approaches, as well as potential novel AD mechanisms revealed by these analyses. 

## 2. Proteomic Characterization of AD Amyloid Plaques and Neurofibrillary Tangles by Laser Capture Microdissection

Extracellular amyloid plaques consist of aggregated Aβ peptides entangled with microglial, neuronal, and vasculature components. Intracellular neurofibrillary tangles are also complex structures marked by anti-tau and anti-ubiquitin immunohistochemistry (IHC) [27,28]. The antibody-based IHC method is a targeted approach for detecting known aggregated proteins in the plaques and NFT of brain tissue, but the exact composition of the aggregated structures could not be uncovered.

**Figure 1:**
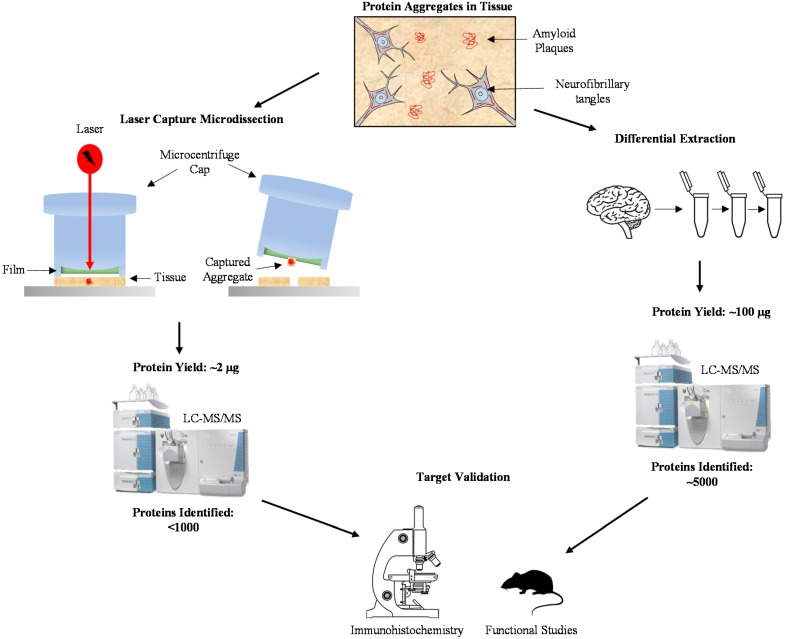
Methods for profiling the aggregated proteome in Alzheimer’s Disease (AD). Isolation of protein aggregates in AD brain can be accomplished using laser capture microdissection or differential extraction. Laser capture microdissection specifically captures protein aggregates, resulting in a protein yield of around 2 µg for 1000 plaques. Using this minute amount, less than 1000 proteins were identified using LC-MS/MS. Differential extraction, the process of isolating insoluble aggregates through repeated centrifugation in varying reagents, yields around 100 µg of protein and around 5000 proteins identified using LC-MS/MS. Regardless of the method of aggregate isolation, protein targets need to be validated using specific immunohistochemical techniques and their function can be determined using comparable research models.

Integration of laser capture microdissection (LCM) [29] with highly sensitive mass spectrometry (MS) [30] enables direct dissection of protein components in these AD aggregated structures [31,32]. In a pioneer study, Liao et al. isolated thioflavin-S-labeled senile plaques from frozen sections of human post-mortem brain tissue, and compared the plaque protein composition with the non-plaque regions by label-free quantification [33]. The analysis was performed with nanoscale liquid chromatography-tandem mass spectrometry (LC-MS/MS) on an LCQ ion trap mass spectrometer, identifying 488 proteins in the isolated plaques, in which 26 proteins were significantly enriched in the plaques compared to non-plaque regions. These proteins were classified into a variety of functional groups, including cell adhesion, cytoskeleton and membrane trafficking, chaperones and inflammation, kinase/phosphatase and regulators, and proteolysis, consistently with diverse cellular components in the plaque area [33]. Notably, the membrane trafficking protein dynein was enriched in the isolated plaques, and its localization was further validated by IHC in a transgenic AD mouse model. This study demonstrates the feasibility of proteomic analysis of minute amounts of LCM-isolated AD samples (Figure 1). 

LCM was also used to isolate NFTs in AD brain for proteomic analysis. Wang et al. isolated NFTs from AD hippocampus samples and performed LC-MS/MS to determine NFT-associated proteins [34]. Out of 155 identified proteins, 63 novel proteins were found to be associated with NFT, including glyceraldehyde-3-phosphate dehydrogenase (GAPDH). The association of GAPDH with NFT was further supported by immunohistochemistry in AD brain samples, as well as biochemical fractionation of detergent-insoluble samples of AD brain lysate.

More recently, Drummond et al. implemented a method to extract proteins from archived, formalin-fixed paraffin-embedded (FFPE) human tissue slides, and analyzed amyloid plaques and NFT from FFPE AD brain tissue using LCM-LC-MS/MS [35]. The FFPE samples were extracted by formic acid and deparaffinized, followed by protein digestion. Using an Orbitrap Q-Exactive mass spectrometer, the group analyzed approximately 900 proteins in the plaques and 500 proteins in NFT with an FDR of 1%, deepening the understanding of neuropathological hallmarks in AD.

LCM allows for the specific isolation of plaques and NFT tissue which can lead to the identification of hundreds of proteins; however, these proteins only represent the most abundant components in the captured tissue areas. Another major drawback of the use of LCM for plaque and NFT isolation is the minute amount of sample that can be collected. For instance, using 10 μm thick sections to capture amyloid plaques, which are heterogeneous in size and about 60 μm in average diameter [31], the protein yield is approximately 2 ng per plaque and 2 µg from 1000 plaques. To address this drawback, protein differential extraction has been developed to increase the protein yield for deep proteome profiling (Table 1).

## 3. Deep Analysis of Aggregated Proteome in AD by Differential Extraction

Differential extraction has long been used for the enrichment of aggregated proteins in neurodegenerative diseases [36], as exemplified by biochemical purification of Aβ and tau in AD [37,38], α-synuclein in PD [25], and TDP-43 in FTLD-U and ALS [26]. Differential extraction is based on the principle that aggregated proteins usually display low solubility and are thus enriched in the pellet after detergent extraction (e.g., sarkosyl) as a detergent-insoluble fraction (Figure 1) [39].

The insolubility of amyloid plaque and NFT components provides an avenue for isolation and subsequent proteomic characterization. Insoluble aggregates can be isolated from whole homogenates of AD brain through sequential extraction. Gozal et al. isolated detergent-insoluble lysate from the frontal cortex of control, AD, and FTLD cases [40]. Label-free LC-MS/MS quantification identified 512 proteins, in which 11 proteins were significantly elevated in AD compared to FTLD and control cases. As expected, tau, Aβ, apolipoprotein E [41], and serum amyloid P [42] were enriched in the AD samples. The alteration of several proteins including serine protease 15, ankyrin B, and 14-3-3 eta, were validated by immunoblotting analysis. 

Following the pilot study [40], Bai et al. performed a comprehensive profiling of aggregate-enriched, detergent-insoluble fractions from all major neurodegenerative diseases, including AD, PD, FTLD-U, ALS, corticobasal degeneration (CBD), and control samples [39]. To identify if proteins change early in the development of AD, mild cognitive impairment (MCI), a prodromal stage of AD, was also analyzed. This large-scale profiling was based on label-free quantification by gel-enhanced LC-MS/MS (gelLC-MS/MS)–protein separation by 1D SDS gel followed by in-gel digestion and LC-MS/MS, leading to the identification of 4216 proteins. After stringent statistical analysis and manual evaluation, a total of 36 proteins were shown to accumulate in AD. In addition to the known aggregate components such as Aβ, tau, ApoE, and complement proteins, the enriched proteins are involved in Aβ clearance [43], phosphorylation networks [16], synaptic plasticity [44], and mitochondrial regulation [45]. Interestingly, several U1 small nuclear ribonucleoprotein (U1 snRNP) spliceosome subunits (U1-70K and U1A) and the interacting RNA helicase Prp5 [46] were found to be highly increased in AD, leading to a novel U1 snRNP pathology, and implicating RNA splicing dysfunction in AD [39]. In addition to late onset sporadic AD cases, the U1 snRNP components were also found to aggregate in early onset genetic cases (e.g., mutations in APP and PS-1), as well as in trisomy 21 (the APP gene is in chromosome 21) [47].

To track the process of protein insolubility during the course of AD development, Hales et al. continued to quantify the detergent-insoluble brain proteome, and correlated them with Aβ and tau proteins in 35 cases of control, asymptomatic phase of AD (AsymAD), MCI, and AD [48]. Among 2711 proteins, six U1 snRNP subunits (U1-70K, U1A, SmD1, SmD2, SmD3, and SmB) are in the top 10 Aβ-correlated proteins, whereas three U1 snRNP subunits (U1-70K, U1A, and SmD) are also correlated with tau insolubility. These results suggest a possible link of these AD aggregated proteins during disease progression.

## 4. Implication of Disease Mechanisms by Aggregated Proteins in AD

Specifically analyzing the aggregate proteome in AD can be used to identify potential mechanisms of disease progression or development (Table 2). Since Aβ and tau are considered pathological hallmarks of AD, it is expected that these proteins would not only be identified in protein aggregates in AD, but also enriched in the AD aggregates compared to control patient aggregates. Consistently, Aβ and tau proteins are identified in the aggregate proteome in all AD patient samples [33,34,40,48]. While the exact molecular mechanisms of AD remain to be understood, aggregated Aβ can contribute to AD progression through neurotoxic effects including disruption of synaptic communication, free radical production, and disrupted calcium homeostasis [49]. The relationship between tau and Aβ is supported by in vitro studies that show Aβ-induced tau-dependent microtubule dysfunction, synaptic damage, and excitotoxicity [50], as well as in vivo studies that indicate Aβ-induced tau-mediated axonal transport defects [51]. Microtubules are key components of intracellular transport that exhibit reduced stability and subsequent reduced axonal transport in AD [52]. Loss of microtubules in AD has been attributed to aggregated tau-induced polyglutamylation of microtubules [52]. Additionally, Aβ oligomers can trigger tau-induced microtubule decay through elevated intracellular calcium, suggesting that Aβ aggregation may be an upstream event of tau-induced microtubule loss [52]. The loss of microtubules leads to impaired axonal transport which leads to dendritic spine decay and subsequent neuronal dysfunction [53].

Inflammatory proteins, including high-temperature requirement serine protease A1 (HTRA1) and complement C3, were found to associate with Aβ and tau aggregates in AD patient brain samples [39,48]. HTRA1 is a secreted serine protease that can bind tumor growth factor-β proteins, inhibiting their anti-inflammatory actions [54]. The correlation of HTRA1 and aggregated Aβ and tau in AD samples suggests possible upregulation of HTRA1 in AD, which could have implications in the inflammation associated with AD [48]. Complement C3 is released from microglia and is involved in phagocytosis [55]. In AD, Aβ initiates a complement cascade in which C3 production increases leading to phagocytosis of not only Aβ plaques, but also synapses [56]. This aberrant activation of microglia may contribute to the neuronal degeneration and synaptic dysfunction associated with AD. The association of inflammatory proteins with Aβ and tau aggregates in AD brain samples further exemplifies an inflammatory component to AD pathology. 

U1 snRNP subunits (notably, U1-70K, SmD, and U1A) are highly correlated with insoluble tau and Aβ, suggesting a possible role in tau aggregation and AD pathogenesis [39,48]. U1 snRNP protein subunits are coupled with small nuclear RNAs (snRNAs) to form spliceosomes, which remove introns from mRNA transcripts in a process known as mRNA splicing [57]. The identification of multiple U1 snRNP subunits in the detergent-insoluble AD proteome strongly suggests the precipitation of the entire U1 snRNP complex. Indeed, IHC staining indicated tangle-like aggregates of snRNA in AD cases, and transmission electron microscopy showed snRNA co-localization with tau NFT [58]. The aggregation of U1-70K, a U1 snRNP, occurred in the form of cytoplasmic tangles in AD brain slices [39]. This localization was later confirmed using electron microscopy in which immunogold-labeled U1-70K co-localized with structures resembling NFT in AD frontal cortex samples [59]. This abnormal localization and enrichment of a U1 snRNP could play a role in AD. Consistently, deep RNA-sequencing revealed impaired RNA splicing in AD cortical samples [39]. This functional deficit could be the result of aggregation of spliceosome components and a loss-of-function effect in the AD brain [39,48]. 

The aggregation of U1-70K in AD has been confirmed in multiple studies, yet the cause of this abnormal aggregation in AD brain samples is still unclear. The presence of two specific low complexity (LC) domains in U1-70K protein suggests an inherent tendency for U1-70K aggregation [60]. Low complexity domains are repetitive sequences of amino acids that display a tendency to aggregate at high concentrations [61]. Recombinant protein studies concluded that one C-terminal LC domain in U1-70K contributed to its aggregation [59]. In AD brain homogenates, endogenous U1-70K aggregates formed direct interactions with recombinant U1-70K that was prone to aggregation via the incorporation of an LC domain. These results suggest that U1-70K aggregation in AD is the result of both an inherent potential for U1-70K to aggregate and co-aggregate with pre-existing seeds.

In addition to the aggregation hypothesis, the U1-70K loss-of-function may be the result of abnormal cleavage and peptide truncation in AD. Bai et al. showed that U1-70K can be cleaved to generate an N-terminal truncation identified as N40K [62]. This truncation occurred in about 50% of the 17 AD brain samples studied [62]. In these cases, the expression of N40K inversely correlated with the expression of U1-70K [62], suggesting that U1-70K loss-of-function could be due to truncation. Functionally, N40K displayed toxic pro-apoptotic effects in primary rat neurons [62]. 

It should be mentioned that N40K also contains a low complexity domain to form aggregates [59]. More recently, Bishof et al. extended the concept and proposed that a large number of RNA binding proteins containing basic-acidic dipeptide (BAD) domains may co-aggregate in Alzheimer’s disease [74]. It will be highly interesting to further study if these RNA binding proteins contribute to AD pathogenesis.

## 5. Conclusions

Protein aggregation is a hallmark of AD typically associated with Aβ and hyperphosphorylated tau, however, other proteins can also self-aggregate or co-aggregate with amyloid plaques and NFT. Identifying this aggregated proteome could provide insight into the underlying mechanisms of AD development and progression. MS techniques coupled with plaque and NFT isolation allow for the analysis of the aggregate proteome in human AD samples. LCM and detergent-insoluble fractionation techniques have been successfully applied to isolate amyloid plaques and NFTs directly from AD brain samples for MS analysis. These techniques have identified novel aggregate proteins including U1-snRNP, a member of the spliceosome necessary for RNA splicing. Further studies have identified splicing loss-of-function in human AD samples. Additionally, comprehensive RNA-seq analyses from multiple cohorts implicate the role of RNA splicing dysfunction in AD [83]. Although further functional studies are needed to determine the exact role of aggregate-associated proteins in AD, MS proves to be an invaluable tool for dissecting AD pathology and pathogenesis.

## Figures and Tables

**Table 1:** A comparison of approaches for protein aggregate isolation for proteomic profiling.

Technique	Protein Yield	Instruments Required	Number of Proteins Identified *	Advantages	Disadvantages
*LCM*	~2 µg from 1000 plaques	Fluorescent Microscope with Laser Capture capability LC-MS/MS	155–900 [33,34,35]	(1) Precise collection of cellular components (2) Conservation of tissue integrity (3) Cellular region comparison within the same tissue	(1) Small amount of protein recovery (2) Extensive time required for LCM
*Differential fractionation*	1% of total protein input (e.g., 100 µg from 10 mg of tissue)	Centrifuge LC-MS/MS	512–4216 [39,40,49]	(1) A sufficient amount of protein can be extracted from individual samples (2) Flexible extraction methods using different combinations of detergents	(1) Detergent soluble aggregate proteins may not be included in the MS analysis (2) Contamination of the aggregated proteome by other detergent insoluble components

* The number of proteins identified may increase with the use of more sensitive instrumentation.

**Table 2:** A comparison of significant AD-specific proteins identified in the insoluble fractions collected from two differential fractionation LC-MS/MS studies.

Protein	GeneBank™ Accession Number	Association with AD
Identified by Bai, B., et al., PNAS, 2013 [39]		
Collagen Type XXV, alpha 1 isoform 2	NP_000032.1	[63]
Cellular retinoic acid binding protein	NP_004369.1	[48]
Dystrobrevin alpha	NP_009224.2	[48]
Complement component 4a preproprotein	NP_116757.2	[64]
Complement component 3	NP_000055.2	[65]
Cyclin G-associated kinase	NP_005246.2	Not Found
Protein tyrosine phosphatase, zeta1	NP_002842.2	[66]
T-cell activation protein phosphatase 2C	NP_644812.1	Not Found
Synaptojanin 1	NP_982271.1	[67]
Amphiphysin	NP_001626.1	[68]
Syntaxin binding protein 5	NP_640337.3	[69]
Regulating synaptic membrane exocytosis 1	NP_055804.2	Not Found
Neuroblastoma-amplified protein (with a Sec39 domain)	NP_056993.2	Not Found
Glutamate receptor interacting protein 1	NP_066973.1	[70]
Mitochondrial nicotinamide nucleotide transhydrogenase	NP_892022.2	[71]
Mitochondrial NFS1 nitrogen fixation 1	NP_066923.3	Not Found
Mitochondrial fumarate hydratase	NP_000134.2	[72]
Optic atrophy 1	NP_570847.1	[73]
Mitochondrial processing peptidase	NP_004270.2	Not Found
U1 small nuclear ribonucleoprotein 70 kDa	NP_003080.2	[74]
U1 small nuclear ribonucleoprotein A	NP_004587.1	[39]
ATP-dependent RNA helicase DDX46, Prp5	NP_055644.2	Not Found
4-Aminobutyrate aminotransferase	NP_001120920.1	[75]
10-Formyltetrahydrofolate dehydrogenase	NP_036322.2	Not Found
Phytanoyl-CoA dioxygenase domain containing protein 1	NP_001094346.1	Not Found
Nicotinamide nucleotide adenylyltransferase 3	NP_835471.1	[76]
Asparagine-linked glycosylation 2	NP_149078.1	Not Found
GTPase activating protein and VPS9 domains 1	NP_056450.2	[77]
Phosphatidylinositol-dependent Rac exchanger 1	NP_065871.2	Not Found
Aminophospholipid transporter	NP_006086.1	[78]
RAN binding protein 16 (exportin 7)	NP_055839.3	[79]
ALFY, involved in macroautophagy	NP_055806.2	Not Found
Identified by Gozal, Y., et al., J. Proteome Res., 2009 [40]		
serum amyloid P component precursor	NP_001630.1	[42]
serine protease 15	NP_004784.2	Not Found
14-3-3, eta polypeptide	NP_003396.1	Not Found
14-3-3, zeta polypeptide	NP_663723.1	Not Found
ankyrin B	NP_066187.2	Not Found
dynamin 1	NP_004399.2	[80]
aquaporin 1	NP_000376.1	[81]
Identified in both studies		
Apolipoprotein E	NP_000032.1	[41]
Microtubule-associated protein tau	NP_058519.2	[16]
Amyloid β peptide	NP_000475.1	[82]
Complement component 4b	NP_001002029.3	[64]
